# Polymeric Caffeic Acid Acts as an Antigen Delivery Carrier for Mucosal Vaccine Formulation by Forming a Complex with an Antigenic Protein

**DOI:** 10.3390/vaccines12050449

**Published:** 2024-04-23

**Authors:** Rui Tada, Yuzuho Nagai, Miki Ogasawara, Momoko Saito, Akihiro Ohshima, Daisuke Yamanaka, Jun Kunisawa, Yoshiyuki Adachi, Yoichi Negishi

**Affiliations:** 1 Department of Drug Delivery and Molecular Biopharmaceutics, School of Pharmacy, Tokyo University of Pharmacy and Life Sciences, 1432-1 Horinouchi, Hachioji 192-0392, Tokyo, Japan; yuzuho21@gmail.com (Y.N.); miki.ogasawara.0012012@gmail.com (M.O.); momomo3p@hotmail.co.jp (M.S.); ba.aky@docomo.ne.jp (A.O.); negishi@toyaku.ac.jp (Y.N.); 2 Laboratory for Immunopharmacology of Microbial Products, School of Pharmacy, Tokyo University of Pharmacy and Life Sciences, 1432-1 Horinouchi, Hachioji 192-0392, Tokyo, Japan; ymnk@toyaku.ac.jp (D.Y.); adachi@toyaku.ac.jp (Y.A.); 3 Laboratory of Vaccine Materials and Laboratory of Gut Environmental System, Microbial Research Center for Health and Medicine, National Institutes of Biomedical Innovation, Health and Nutrition (NIBIOHN), 7-6-8 Saito-Asagi, Ibaraki 567-0085, Osaka, Japan; kunisawa@nibiohn.go.jp

**Keywords:** polyphenol, antigen delivery, mucosal vaccine, mucosal adjuvant

## Abstract

The development of mucosal vaccines, which can generate antigen-specific immune responses in both the systemic and mucosal compartments, has been recognized as an effective strategy for combating infectious diseases caused by pathogenic microbes. Our recent research has focused on creating a nasal vaccine system in mice using enzymatically polymerized caffeic acid (pCA). However, we do not yet understand the molecular mechanisms by which pCA stimulates antigen-specific mucosal immune responses. In this study, we hypothesized that pCA might activate mucosal immunity at the site of administration based on our previous findings that pCA possesses immune-activating properties. However, contrary to our initial hypothesis, the intranasal administration of pCA did not enhance the expression of various genes involved in mucosal immune responses, including the enhancement of IgA responses. Therefore, we investigated whether pCA forms a complex with antigenic proteins and enhances antigen delivery to mucosal dendritic cells located in the lamina propria beneath the mucosal epithelial layer. Data from gel filtration chromatography indicated that pCA forms a complex with the antigenic protein ovalbumin (OVA). Furthermore, we examined the promotion of OVA delivery to nasal mucosal dendritic cells (mDCs) after the intranasal administration of pCA in combination with OVA and found that OVA uptake by mDCs was increased. Therefore, the data from gel filtration chromatography and flow cytometry imply that pCA enhances antigen-specific antibody production in both mucosal and systemic compartments by serving as an antigen-delivery vehicle.

## 1. Introduction

Infectious diseases pose a significant risk, and despite advances in modern medicine, they remain challenging to eliminate. Currently, infectious diseases rank as the second most common cause of death globally [1,2]. The Coronavirus Disease 2019 (COVID-19) pandemic has highlighted the severe repercussions of highly virulent and contagious pathogenic microorganisms on a global scale. Although the creation of innovative RNA vaccinations and improvements in vaccination initiatives have been made, the number of new cases did not significantly decline worldwide during the pandemic [3,4]. Conventional vaccine techniques that involve introducing substances beneath the skin or within the muscle tissue have not yet been successful in eradicating the pandemic. One possible reason for this is their inability to stimulate immune responses within the mucous membranes, including the generation of immunoglobulin A (IgA), which targets particular pathogens [5]. Given that current vaccines have not been effective in preventing infection, particularly in regard to the initial colonization of mucosal surfaces by pathogens [6], developing a robust vaccine that elicits antigen-specific mucosal immune responses is essential for the preparation of the next pandemic [7].

Mucosal vaccination is widely regarded as the most effective approach for combating infectious diseases caused by pathogens. Recent advancements in mucosal vaccines have demonstrated their ability to provide superior protection against both systemic and mucosal infections, which are the primary entry points for most pathogenic microorganisms [8,9,10].

Despite the fact that mucosal vaccines have demonstrated greater effectiveness than conventional parenteral vaccines, only a limited number of live-attenuated and inactivated mucosal vaccines have been authorized for clinical use [11]. Considering that both forms of mucosal vaccines contain entire pathogenic microorganisms, there exists a persistent threat of unforeseen adverse consequences attributable to the toxicity and antigenicity of these pathogens. In order to effectively address this issue, the development of subunit mucosal vaccines that exclusively contain a microbial antigen is essential for the widespread implementation of mucosal vaccine systems in clinical settings. Subunit mucosal vaccines offer several advantages over live-attenuated or inactivated vaccines in terms of their ability to elicit a targeted immune response with fewer adverse effects. One of the key benefits of these vaccines is that they can be customized to incorporate specific antigens that trigger a protective immune response while avoiding the need to include the entire pathogen. This customization allows for a more targeted and effective approach to vaccine development [12]. Unfortunately, there are currently no vaccines of this kind that have been approved because of the lack of a proven and dependable method for stimulating mucosal immune responses in hosts using mucosal adjuvants [13,14] or for delivering microbial antigens to mucosal dendritic cells (mDCs) using antigen-delivery vehicles [15,16]. The primary cause of this situation is the insufficient ability of the mucosa to respond to antigenic proteins, which is the result of the immune system’s tolerance to foreign antigens [11,17,18,19]. In addition, mucosal epithelial cells establish tight junctions that hinder the passage of macromolecules, such as antigens, across the physical barrier. This prevents access to mDCs situated in the lamina propria, which is essential for initiating mucosal immune responses [20]. A safer and more efficient method is necessary to stimulate antigen-specific immune responses in mucosal areas in order to develop successful mucosal vaccines.

Polyphenols, also known as phenolic compounds, are substances that occur naturally in plants, such as fruits, vegetables, and coffee. These compounds are consumed in significant amounts in our daily lives, with some people consuming up to 1 g per day [21,22]. In recent years, polyphenols have received considerable attention because of their potential health benefits. Research has demonstrated that the daily consumption of polyphenols can aid in preventing cardiovascular disease, cancer, and infectious diseases [23,24,25]. There are numerous sources of polyphenols, and their biological activities have been extensively studied. Traditionally, polyphenols are known for their antioxidant properties [26,27,28], but recent research suggests that they also have immunomodulatory effects on various immune cells. Polyphenols, particularly those with high molecular weights, like lignin, which is produced through the enzymatic polymerization of phenolic compounds, have been discovered to possess antiviral and antibacterial properties that bolster the immune system [29,30,31].

Our research has focused on the immune-regulating properties of lignin-like polyphenols that were generated in vitro through the use of horseradish peroxidase and phenylpropanoids. We have been exploring these effects for an extended period of time. Recently, it was discovered that intranasal immunization with an antigenic protein and enzymatically polymerized caffeic acid (pCA) induced specific antibody responses in both systemic and mucosal compartments in mice. This makes pCA a promising mucosal adjuvant for nasal vaccines against infectious diseases [32,33,34]. However, the molecular mechanisms underlying pCA’s mucosal adjuvant properties are still unclear. Adjuvants generally enhance the uptake and presentation of antigens to antigen-presenting cells (APCs), particularly dendritic cells (DCs), and/or activate the innate immune system [35]. Our study aimed to uncover the mechanism responsible for the enhanced production of antigen-specific antibodies after the intranasal administration of pCA. Specifically, we investigated whether pCA not only activates innate immune responses at the administration site but also acts as an antigen-delivery vehicle, facilitating antigen delivery to the lamina propria, where mDCs are located.

## 2. Materials and Methods

### 2.1. Mice

Six-week-old female BALB/cCrSlc mice were procured from Japan SLC in Shizuoka, Japan, and kept under specific pathogen-free (SPF) conditions throughout the study. All experimental procedures involving animals were conducted using mice aged between 7 and 10 weeks. The animal experimental procedures were approved by the Tokyo University of Pharmacy and Life Sciences Committee for Laboratory Animal Experiments, and the approval numbers for these procedures were P16-12, P17-26, P18-71, and P19-58.

### 2.2. Materials

Sigma-Aldrich (St. Louis, MO, USA) was the supplier of horseradish peroxidase (HRP), and 3-(3,4-dihydroxyphenyl)-2-propenoic acid (also known as caffeic acid; CA) was obtained from Tokyo Chemical Industry Co., Ltd. (Tokyo, Japan). Low endotoxin egg white ovalbumin (OVA) (less than 1 EU/mg) and cholera toxin were procured from FUJIFILM Wako Pure Chemical Corporation (Osaka, Japan). Additionally, Thermo Fisher Scientific (Waltham, MA, USA) was the source of Alexa Fluor^TM^ 488-conjugated OVA (AF488-OVA).

### 2.3. Preparation of a Polymeric Caffeic Acid

A lignin-like polymeric caffeic acid was synthesized through oxidative polymerization, as described in our previous study [36]. The synthesis was initiated by neutralizing 200 mg of caffeic acid with sodium hydroxide and diluting the resulting solution to 10 mL with phosphate-buffered saline containing 1 mg of horseradish peroxidase (HRP). Next, we added 1.5 molar equivalents of hydrogen peroxide dropwise to the mixture while stirring for 1 h at 25 °C. The mixture was stirred for an additional two hours at 25 °C and then heated to 100 °C for 20 min to inactivate and precipitate the HRP. After centrifugation (12,000 rpm, 4 °C), the supernatant was collected and dialyzed against deionized water for two days using a regenerated cellulose membrane with a molecular weight cut-off of 50,000 (Repligen, Waltham, MA, USA). This process was followed by lyophilization to obtain the polymeric caffeic acid (pCA). We tested the pCA preparation for endotoxin contamination using an Endospecy ES-50 M kit from Seikagaku Biobusiness Corporation (Tokyo, Japan), and the results showed a very low endotoxin content of 231.5 pg/mg. To prepare a stock solution (10 mg/mL), we dissolved all samples in endotoxin-free phosphate-buffered saline (PBS; pH 7.4) from FUJIFILM Wako Pure Chemical Corporation and filtered the solution through 0.45 μm filter membranes from Osaka Chemical Co., Ltd. (Osaka, Japan) to remove any contaminants. The stock solution was then stored at −20 °C until use.

### 2.4. Expression of the Genes Induced by pCA at Nasal Mucosa

Mice were administered a mix of medetomidine (0.75 mg/kg), midazolam (4 mg/kg), and butorphanol tartrate (5 mg/kg) via intraperitoneal injection to induce anesthesia. Afterward, they were administered either PBS or pCA (100 µg/mouse) nasally, with a volume of 13 µL (6.5 µL per nostril). The mice were later euthanized by cervical dislocation, and their nasal tissues and spleens were collected for the examination of gene expression changes resulting from intranasal pCA administration. RNA was extracted from the nasal tissue using the FavorPrep Tissue Total RNA Mini Kit (Favorgen Biotech Corporation, Ping-Tung, Taiwan) and treated with DNase I (Roche Life Science, Penzberg, Germany) to ensure purity. The RNA concentration was measured using spectrophotometry. Complementary DNA (cDNA) was synthesized using the ReverTra Ace qPCR RT Master Mix (Toyobo, Tokyo, Japan) following the manufacturer’s instructions. qRT-PCR was performed on a CFX Connect Real-Time PCR Detection System (Bio-Rad, Hercules, CA, USA) using Thunderbird SYBR qPCR Mix (Toyobo) and the primers synthesized by Eurofins Genomics (Tokyo, Japan). For this research, we chose beta-2-microglobulin (B2M) as the housekeeping gene for normalization. B2M is well-established as a dependable reference gene in a variety of tissues and experimental conditions. Previous studies, including our own, have successfully utilized B2M as a housekeeping gene [32]. The expression of genes of interest was normalized to the reference gene B2M, and the relative gene expression among samples was calculated using the comparative Ct (ΔΔCt) method. Melting curve analysis was performed to confirm specific amplification, with the qRT-PCR conditions including an initial step at 95 °C for 1 min, followed by 40 cycles at 95 °C for 15 s and 60 °C for 1 min. The primer pairs used in the experiment are as follows: IL-1α, 5′-ACGTCAAGCAACGGGAAGATTC-3′ (forward) and 5′-ATGGGTTATGGACTGCAGGTC-3′ (reverse); IL-1β, 5′-TACAAGGAGAACCAAGCAACGAC-3′ (forward) and 5′-TGCCGTCTTTCATTACACAGGAC-3′ (reverse); IL-6, 5′-GAAATGATGGATGCTACCAAACTG-3′ (forward) and 5′-CTCTCTGAAGGACTCTGGCTTTG-3′ (reverse); GM-CSF, 5′-TGGGCATTGTGGTCTACAGC-3′ (forward) and 5′-GCGGGTCTGCACACATGTTA-3′ (reverse); TNF-α, 5′-ACCGTCAGCCGATTTGCTATC-3′ (forward) and 5′-ATGGGCTCATACCAGGGTTTG-3′ (reverse); CCL3, 5′-ACCATGACACTCTGCAACCAAG-3′ (forward) and 5′-CAACGATGAATTGGCGTGGAATC-3′ (reverse); TGF-β, 5′-TACGCCTGAGTGGCTGTCTTTTG-3′ (forward) and 5′-CGTGGAGTTTGTTATCTTTGCTGTC-3′ (reverse); IFN-β1, 5′-TTACACTGCCTTTGCCATCC-3′ (forward) and 5′-ACTGTCTGCTGGTGGAGTTCAT-3′ (reverse); IL-12p40, 5′-TGGTTTGCCATCGTTTTGCTG-3′ (forward) and 5′-ACAGGTGAGGTTCACTGTTTCT-3′ (reverse); β2-microglobulin (B2M), 5′-TTCTGGTGCTTGTCTCACTGA-3′ (forward) and 5′-CAGTATGTTCGGCTTCCCATTC-3′ (reverse). The CFX manager software version 3.1 (BIO-RAD) was used to analyze the data, and cycle threshold (Ct) values were calculated. The results are expressed as relative gene expression levels normalized to B2M.

### 2.5. Assessment of Complex Formation of pCA and Antigen

The formation of complexes between pCA and OVA was determined by conducting an analysis using size-exclusion chromatography (SEC). A mixture of pCA and OVA was prepared in a mass ratio of 1:10 and allowed to stand at 25 °C for 30 min. Subsequently, the mixture was subjected to SEC. After the incubation process, the solution was passed through a filter membrane with a pore size of 0.45 µm to eliminate any particulate matter that was insoluble. High-performance liquid chromatography (HPLC) was used for this analysis with a size-exclusion column (OHpak SB-803 HQ column, 8.0 × 300 mm; Resonac Corporation, Tokyo, Japan). Throughout the experiment, PBS was used as the mobile phase, with a flow rate of 0.5 mL/min. Eluted compounds were detected using a UV detector set at a wavelength of 280 nm.

### 2.6. Flow Cytometric Analysis of Antigen Uptake

Six hours after intranasally administering 50 μg/mouse AF488-OVA and 100 μg/mouse pCA to mice, single-cell suspensions were obtained from nasal tissues to analyze the uptake of AF488-OVA by dendritic cells (DCs) in the nasal mucosa. The nasal tissues were homogenized in PBS and centrifuged. To obtain single-cell suspensions, red blood cells were lysed with RBC lysis buffer (BioLegend, San Diego, CA, USA). The cells were then washed with staining buffer (PBS containing 2% heat-inactivated fetal bovine serum (FBS; Biowest, Nuaillé, France) and 0.1% sodium azide) and blocked with anti-mouse CD16/CD32 (clone 2.4G2, Tonbo Biosciences, San Diego, CA, USA) for 20 min on ice to block Fc receptors. After being washed with staining buffer, cells were stained with PE/Cy7 anti-mouse CD45 (clone 30-F11, BioLegend) in combination with APC anti-mouse CD11c (clone N418, BioLegend) or an isotype control for 30 min on ice. Finally, the samples were analyzed using a FACSCanto instrument (BD Biosciences, San Jose, CA, USA).

### 2.7. Statistics

Welch’s correction was employed to conduct an unpaired *t*-test and determine statistical differences. The significance level was set at 0.05, and all data were analyzed using GraphPad Prism 9 software (GraphPad Software, La Jolla, CA, USA).

## 3. Results

### 3.1. Expression of Genes Related to the Mucosal Immune Responses of Mice That Received Nasal Administration of pCA

**Figure 1:**
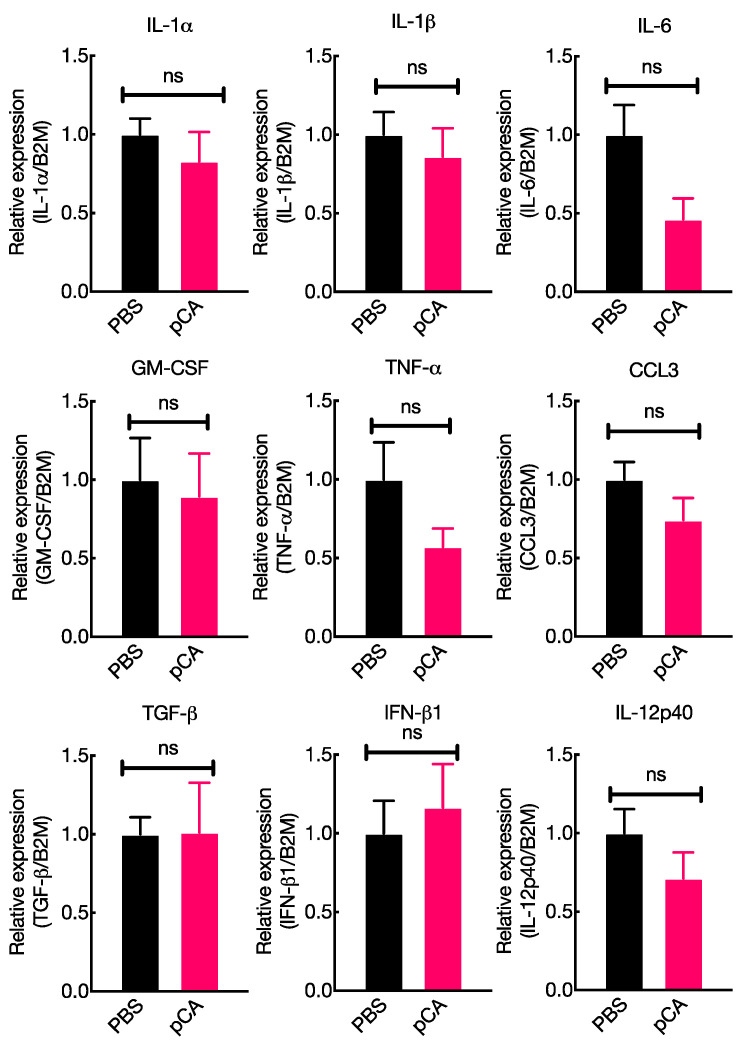
Gene expression associated with the development of antigen-specific IgA responses in mice following the intranasal administration of pCA. Nasal tissues were obtained from mice that received either PBS or pCA (100 µg/mouse) and harvested 16 h after administration. Total RNA was extracted and cDNA synthesized, and gene expression changes were determined using quantitative reverse transcriptase-polymerase chain reaction (qRT-PCR). The average ± standard deviation (SD) values of three biological replicates were reported. The experiments were independently repeated three times. Statistical differences were assessed using an unpaired *t*-test with Welch’s correction, and a *p*-value of less than 0.05 was considered statistically significant. ns: not significant (*p* > 0.05), * *p* < 0.05.

Typically, the induction of antigen-specific immune responses involves the activation of the innate immune system and/or enhancement of antigen uptake and presentation by APCs, such as DCs [35]. Soluble factors, such as cytokines, secreted by both immune and non-immune cells typically regulate immune responses to antigens [37,38,39]. Previous studies have shown that pCA induces the secretion of cytokines by various immune cells [36,40,41]. We aimed to investigate the mechanisms underlying the mucosal adjuvant activity of pCA by identifying genes involved in innate immune responses that have the potential to enhance mucosal IgA responses (interleukin (IL-1)α, IL-1β, IL-6, granulocyte-macrophage colony-stimulating factor (GM-CSF), TNF-α, C-C motif chemokine ligand 3 (CCL3), transforming growth factor-β (TGF-β), IFN-β1, and IL-12p40) following the intranasal administration of pCA. The results shown in Figure 1 demonstrate that the intranasal administration of pCA did not stimulate the expression of genes associated with innate immune responses that could potentially enhance mucosal IgA responses in the nasal region [42,43,44,45,46,47,48]. These results clearly indicated that the activation of mucosal immunity through the activation of innate immune responses did not occur as a result of pCA administration.

### 3.2. pCA Forms Complexes with Antigenic Proteins

**Figure 2:**
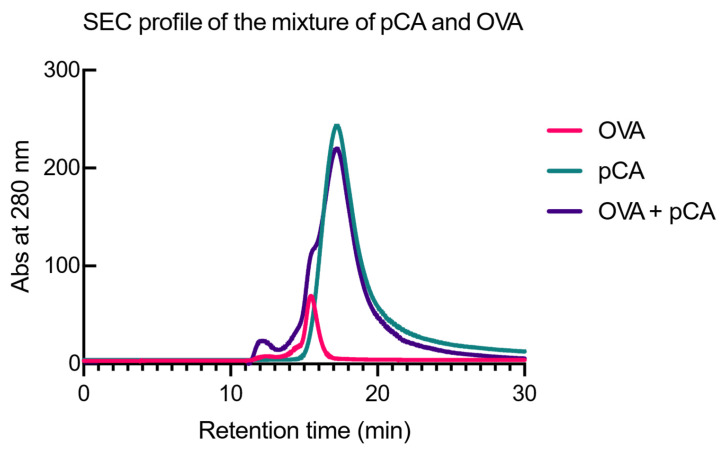
Gel filtration chromatography analysis examining the pCA complex formation with OVA. The OHpak SB-803 HQ column (8.0 × 300 mm) was pre-equilibrated with PBS and eluted with PBS at a flow rate of 0.5 mL/min. The samples, comprising OVA alone, pCA alone, or a mixture of pCA and OVA incubated at room temperature, were injected into the column and monitored using a UV detector set to 280 nm.

For the induction of antigen-specific mucosal immune responses, it is crucial that the antigen be phagocytosed by antigen-presenting cells, such as mucosal dendritic cells, which are located in the lamina propria underlying the mucosal epithelial layers. Thus, improving the efficiency of antigen delivery to mucosal dendritic cells is critical for the development of mucosal vaccines [49,50]. To achieve this, it is essential that pCA functions as an antigenic protein carrier; that is, pCA interacts with antigenic proteins to form complexes. Therefore, we investigated the potential of pCA to form complexes with antigenic proteins by size-exclusion chromatography (SEC). Figure 2 shows the SEC chromatograms of pCA and OVA separately and their mixtures. The SEC chromatogram showed monomeric peaks eluting at 17.2 and 15.5 min, representing pCA and OVA, respectively. In addition, the SEC chromatogram of the pCA–OVA mixture showed a distinct new elution peak at 12.2 min, indicating the formation of the pCA–OVA complex.

### 3.3. pCA Promotes the Delivery of Antigenic Proteins to Nasal DCs

**Figure 3:**
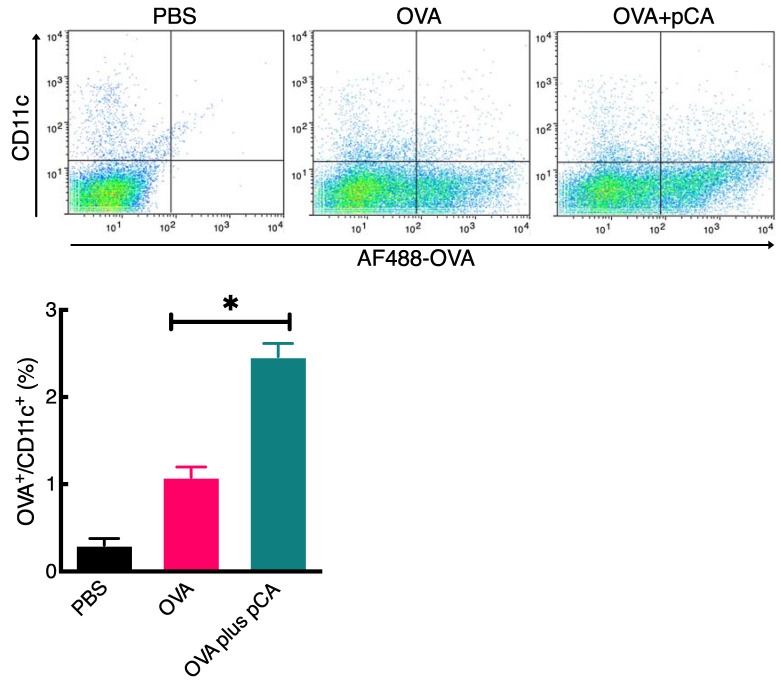
The uptake of OVA by nasal CD11c^+^ dendritic cells (DCs) following the intranasal administration of OVA with pCA. To investigate this, female BALB/c mice were immunized intranasally with PBS, OVA alone (50 μg/mouse), or OVA with pCA (50 μg/mouse and 100 µg/mouse, respectively). After 6 h, single-cell suspensions were isolated from the nasal tissues and treated with anti-mouse CD11c mAb or isotype control for staining. The uptake of AF488-OVA was evaluated using flow cytometry, with four mice in each group used for analysis. A *t*-test with Welch’s correction was used to determine significance, and the results showed a *p*-value of less than 0.05. * *p* < 0.05.

The successful delivery of antigenic proteins to antigen-presenting cells (APCs) is crucial for inducing antigen-specific immunity in the host. In this study, we examined the effect of pCA on the uptake of AF488-OVA by nasal dendritic cells (DCs). Our flow cytometric analyses, as shown in Figure 3, revealed a significant increase in the proportion of AF488^+^ cells among the CD11c^+^ cells in the DC population of mice receiving both AF488-OVA and pCA (2.5 ± 0.30%) compared with those receiving AF488-OVA alone (1.1 ± 0.24%). This suggests that the pCA-enhanced uptake may serve as a mechanism for inducing both mucosal and systemic antigen-specific immune responses.

## 4. Discussion

The scientific community has prioritized the development of safe and efficient vaccines that can protect against infectious diseases, particularly after the global pandemic caused by severe acute respiratory syndrome coronavirus 2 (SARS-CoV-2). This study aimed to explore the potential of enzymatically polymerized caffeic acid (pCA) as a mucosal adjuvant for nasal vaccines against infectious diseases. Prior research has suggested that pCA, along with other enzymatically polymerized polyphenols, can enhance antigen-specific immune responses when administered intranasally with antigenic proteins. However, the precise molecular mechanisms underlying the mucosal adjuvant effects of pCA remain unidentified.

The study findings, which reveal that pCA forms complexes with antigenic proteins and enhances antigen uptake by mucosal dendritic cells, provide insights into the molecular mechanisms underlying pCA’s function as a promising adjuvant for mucosal vaccines. The formation of complexes between pCA and antigens may facilitate the antigen passage through the mucosal barrier and promote antigen delivery to mucosal dendritic cells, which is crucial for inducing mucosal immune responses. Additionally, the results suggest that pCA may directly enhance antigen uptake and presentation by mucosal dendritic cells. Future research is anticipated to elucidate the detailed mechanisms by which the pCA–antigen complex formation contributes to the induction of mucosal immune responses.

This study aimed to investigate the mechanisms underlying the mucosal adjuvant effects of pCA. Adjuvants typically work through two mechanisms: activating the innate immune system and improving antigen uptake and presentation to APCs, particularly DCs [35]. Additionally, previous research has shown that pCA has immunomodulatory properties. The oral administration of pCA has been found to activate NK cells and induce cytokine production in mouse splenocytes in vitro [36,40,41,51]. Therefore, it is believed that pCA enhances antigen-specific immune responses by activating innate immunity in the mucosa. However, contrary to our initial expectations, the intranasal administration of pCA did not stimulate the expression of genes associated with innate immune responses that could potentially enhance mucosal IgA responses in the nasal region (Figure 1). Next, we investigated the potential of pCA as a delivery vehicle for antigenic proteins in mucosal dendritic cells. Recent studies have shown that polyphenols can effectively interact with proteins through non-covalent intermolecular forces, such as hydrogen bonding, hydrophobic interactions, van der Waals forces, and electrostatic interactions. These interactions result in the formation of soluble protein–polyphenol complexes, which can affect the bioavailability and properties of both components [52,53,54,55]. In our SEC analysis, we observed that the mixture of pCA and OVA eluted faster than the individual components, indicating the formation of a complex between pCA and OVA (Figure 2). Moreover, pCA acted as an antigen-delivery vehicle, facilitating antigen delivery to mDCs (Figure 3). This could potentially lead to an enhanced production of antigen-specific antibodies in both systemic and mucosal compartments. However, the study has some limitations. It is not clear how pCA enhances the delivery of antigenic proteins to mDCs in the lamina propria. While it is known that low-molecular-weight polyphenols can cross the small intestine without mucosal absorption and are transported by active transport or sodium-dependent glucose transport [56,57,58,59,60], high-molecular-weight polyphenols have limited tissue penetration due to their polymeric and hydrophilic nature [61]. Nevertheless, given that pCA has been shown to activate various immune cells in vitro [40,41,51,62], it is possible that there is an unknown transport pathway involving specific receptors. Further research is needed to understand the molecular mechanisms involved. Additionally, the observed activation of splenocytes and dendritic cells by pCA, leading to cytokine production, suggests a potential interaction with dendritic cell membrane receptors [36]. This interaction could facilitate antigen uptake and presentation, a hypothesis supported by our previous research indicating enhanced dendritic cell uptake through the complex formation between pCA and antigens. Identifying the specific receptors and pathways involved in pCA’s immunomodulatory effects remains a critical area for future investigation.

This research highlights the potential of pCA as an antigen delivery carrier, but it is important to acknowledge certain limitations. Firstly, the model antigen OVA utilized in this study may not be comparable to actual pathogenic antigens. Future studies must investigate the mucosal adjuvant effects of pCA using disease-specific antigens. Moreover, preclinical trials involving human cells or tissues, along with a comprehensive safety profile assessment, will be necessary for clinical application. Furthermore, to gain a deeper insight into the mechanisms underlying pCA’s mucosal adjuvant effects, it is crucial to examine the influence of the pCA–antigen complex formation on mucosal immune responses at the molecular and cellular levels.

The results of this study have potential implications for the development of safe and effective mucosal vaccines against various infectious diseases. The use of pCA as a mucosal adjuvant could greatly enhance the efficacy of mucosal vaccines by facilitating antigen delivery to nasal DCs while minimizing unexpected side effects due to pathogen toxicity and antigenicity. Further preclinical and clinical studies on the safety and efficacy of pCA are necessary to develop novel mucosal vaccines that can effectively prevent infectious diseases.

The mucosal immune system is a vital initial line of defense against pathogens that enter through mucosal surfaces, such as the respiratory and gastrointestinal tracts. The development of effective mucosal vaccines has been a long-standing challenge due to the unique features of the mucosal immune system, including the presence of physical and chemical barriers, the complexity of mucosal antigen presentation, and the induction of tolerance. The use of mucosal adjuvants, such as pCA, represents a promising strategy to overcome these challenges and enhance the efficacy of mucosal vaccines. By facilitating antigen delivery to mucosal dendritic cells and potentially modulating immune responses, mucosal adjuvants can help to break mucosal tolerance and induce robust antigen-specific immunity. The results of this study, demonstrating the capacity of pCA to form complexes with antigenic proteins and enhance the antigen uptake by nasal dendritic cells, provide a strong rationale for further exploring its potential as a mucosal adjuvant. Future studies should investigate the impact of pCA on the induction of mucosal antibody responses, such as secretory IgA, which plays a crucial role in neutralizing pathogens at mucosal surfaces. Additionally, evaluating the ability of pCA to induce long-lasting memory immune responses and its compatibility with different vaccine platforms, such as subunit vaccines or nanoparticle-based vaccines, will be important for its clinical translation.

The importance of this research lies in its showing of pCA’s capacity to serve as a promising adjuvant for mucosal vaccines. The creation of effective mucosal adjuvants is a critical challenge in the development of mucosal vaccines for the prevention of infectious diseases. As an antigen-delivery carrier, pCA has the potential to emerge as a novel mucosal adjuvant capable of effectively inducing mucosal immune responses. Furthermore, pCA is a safe and naturally derived substance, which suggests that the barriers to clinical application may be relatively low. According to the results of this study, the development of mucosal vaccines using pCA is expected to make a significant contribution to the prevention of infectious diseases in the future.

## 5. Conclusions

The findings of this study indicate that enzymatically polymerized caffeic acid (pCA) presents a promising prospect as a mucosal adjuvant for nasal vaccines. The capacity of pCA to form complexes with antigenic proteins and enhance antigen uptake by mucosal dendritic cells suggests its potential to augment the efficacy of mucosal vaccines significantly. In facilitating the delivery of antigens to nasal dendritic cells and potentially improving antigen passage through the mucosal barrier, pCA offers a novel strategy to surmount the limitations of conventional vaccine formulations. As a naturally derived substance, pCA’s favorable safety profile further supports its potential for clinical applications. To fully recognize the potential of pCA as a mucosal adjuvant, future investigations should concentrate on exploring its efficacy using disease-specific antigens and evaluating its safety in human cell or tissue models. Moreover, elucidating the molecular mechanisms underpinning pCA’s immunomodulatory effects will provide invaluable insights into its role in enhancing mucosal immune responses. The development of pCA as a mucosal adjuvant represents a considerable advancement in the field of vaccine development. By harnessing the potential of this innovative approach, we can create safer and more effective mucosal vaccines to prevent a wide array of infectious diseases. The continued research and clinical development of pCA-based mucosal vaccines hold great promise for addressing the global burden of infectious diseases and enhancing public health outcomes worldwide.

## Data Availability

The data presented in this study are available in the article.
